# C-type lectins from the nematode parasites *Heligmosomoides polygyrus* and *Nippostrongylus brasiliensis*

**DOI:** 10.1016/j.parint.2009.08.011

**Published:** 2009-12

**Authors:** Yvonne Harcus, Gavin Nicoll, Janice Murray, Kara Filbey, Natalia Gomez-Escobar, Rick M Maizels

**Affiliations:** Institute of Immunology and Infection Research, University of Edinburgh, UK

**Keywords:** Evolution, Helminth, Lectin, Nematode, Secretion

## Abstract

The C-type lectin superfamily is highly represented in all metazoan phyla so far studied. Many members of this superfamily are important in innate immune defences against infection, while others serve key developmental and structural roles. Within the superfamily, many proteins contain multiple canonical carbohydrate-recognition domains (CRDs), together with additional non-lectin domains. In this report, we have studied two gastrointestinal nematode parasites which are widely used in experimental rodent systems, *Heligmosomoides polygyrus* and *Nippostrongylus brasiliensis*. From cDNA libraries, we have isolated 3 new C-type lectins from these species; all are single-CRD proteins with short additional N-terminal domains. The predicted *Hp*-CTL-1 protein contains 156 aa, *Nb*-CTL-1 191 aa and *Nb*-CTL-2 183 aa; all encode predicted signal peptides, as well as key conserved sequence motifs characteristic of the CTL superfamily. These lectins are most similar to *C. elegans* CLEC-48, 49 and 50, as well as to the lectin domains of mammalian immune system proteins CD23 and CD206. RT-PCR showed that these *H. polygyrus* and *N. brasiliensis* genes are primarily expressed in the gut-dwelling adult stages, although *Nb*-CTL-2 transcripts are also prominent in the free-living infective larval (L3) stage. Polyclonal antibodies raised to *Hp*-CTL-1 and *Nb*-CTL-1 reacted to both proteins by ELISA, and in Western blot analysis recognised a 15-kDa band in secreted proteins of adult *N. brasiliensis* (NES) and a 19-kDa band in *H. polygyrus* ES (HES). Anti-CTL-1 antibody also bound strongly to the cuticle of adult *H. polygyrus*. Hence, live parasites release C-type lectins homologous to some key receptors of the mammalian host immune system, raising the possibility that these products interfere in some manner with immunological recognition or effector function.

## 1 Introduction

The C-type lectin (CTL) superfamily has emerged as one of the largest and most widely expressed set of proteins among metazoan organisms in general [1,2]. They are characterized by a conserved 115–130 amino acid carbohydrate-recognition domain (CRD) which includes multiple Ca^2+^-binding sites, which determine the calcium (C-type) dependency of ligation. They contain 4 critical cysteine residues which stabilise a two-loop structure, with the key residues involved in sugar binding located on a long flexible loop.

CTLs may be secreted or transmembrane proteins, and in the latter case they are classed as type II with an intracellular N-terminus. They often comprise multiple domains which confer higher avidity for their ligand. For example, mammalian macrophage mannose receptor (CD206) contains a tandem array of 8 CRDs [3]. Moreover, additional functions are often incorporated through non-CRD domains, such as the collagenous stalk of collectin proteins [4]. Some members of the gene family have lost lectin activity through evolution—such as the NK receptors Ly49, NKG2, and CD94 NK receptor families, which bind MHC Class I in a Ca^2+^-independent manner; these homologues tend to have more compact structures with shorter loops [5]**.**

CTLs are perhaps most prominent in the mammalian immune system [6,7]. Well-known examples include the selectins which mediate inflammatory extravasation through binding of leukocyte integrins (CD11/CD18) to E-selectin expressed on activated epithelial cells [8]. In serum, soluble C-type lectins such as mannose binding protein (MBP) recognise terminal mannose residues found on nonmammalian glycans, and activate the complement cascade. Additionally, CTLs on the surface of immune cells [9] include CD23, the low affinity Fcε receptor [10], CD206 [3], Dectin-1, L-SIGN and DC-SIGN (CD209) [11], the lattermost mediating endocytosis and breakdown of glycosylated proteins for antigen presentation. In the case of Dectin-1, the CTL contains an intracellular motif allowing it to transduce a cell-activating signal, so that binding of fungal β-glucan can initiate an innate immune response [12].

Helminth parasites can establish long-term infections in mammalian hosts, attributed to their ability to neutralise host defence mechanisms [13,14]. Interestingly, L-SIGN [15], DC-SIGN [16] and other CTL family members [17] recognise schistosome egg glycans, indicating that C-type lectin receptors (CLRs) may constitute an ancient pattern recognition system mediating innate interactions between mammals and helminth parasites. The finding that helminths themselves also encode C-type lectin-like genes [18,19], raised the possibility that they may function to interfere with host immunity [20]. Nevertheless, it is clear that other roles are fulfilled by CTLs within the nematode phylum. Most conspicuously, the free-living nematode *Caenorhabditis elegans* encodes at the genomic level no fewer than 278 CTL-like proteins [21], and several of these have been demonstrated experimentally to be up-regulated and protective in the event of bacterial infection of the worm [22]. Indeed, it now appears clear that the CTL response to microbial challenge is an ancient and conserved system common to worms and mammals. Most remarkable, perhaps, is the role of a CTL (*Mermaid*) from the marine nematode *Laxus oneistus*, which is expressed on the worm surface and binds symbiotic bacteria necessary for the metabolism of the worm [23]. Hence, in nematodes as well as in other contexts such as snake venoms [24], CTLs may fulfill not solely a defensive role, but a broader one in regulating interactions with other organisms, be they pathogens, symbionts, prey or hosts.

Amongst the parasitic nematodes, two of the most widely-studied model systems are *Heligmosomoides polygyrus* and *Nippostrongylus brasiliensis*, natural parasites of mice and rats respectively. These parasites have been studied in particular for their ability to drive Th2 immune responsiveness [25–28], and to modulate host immunity [13,29,30] through the secretion of immunomodulatory proteins [31–33]. In this report we identify new C-type lectins from these rodent parasites which, as we show, are preferentially expressed by the mature, gut-dwelling adult stages and form part of the parasite secretions presumed to mediate immunological interactions with the host.

## 2 Materials and methods

### 2.1 *H. polygyrus* life cycle

The gastrointestinal nematode parasite *H. polygyrus bakeri* (syn. *Nematospiroides dubius*) was maintained as originally described elsewhere [34]. Briefly, CF1 mice were infected with 400–600 infective stage larvae by gavage, and maintained in individually-ventilated cages for up to 28 days. Faecal pellets were collected from day 10 onwards, and plated with activated charcoal to provide culture conditions for eggs to hatch and larvae to grow over a 9-day period to the infective larval (L3) stage. Larvae were collected from plates and washed 3 times in water. Larvae were stored for up to 60 days at 4 °C before use. Adult worms were collected 14–28 days post-infection by Baermannization and used immediately for mRNA isolation or in vitro culture for secreted antigens.

### 2.2 *H. polygyrus* cDNA library construction and identification of *Hp*-CTL-1

A Uni-ZAP XR cDNA phage library was constructed from approximately 400 µl packed volume of adult worms taken 28 days post-infection. Following RNA extraction in TriZOL and DNase treatment, the poly-A+ mRNA fractions were isolated using an Ambion Micropoly(A) Purist kit. First strand cDNA synthesis was primed from an oligo-d(T) primer with an unmethylated *Xho*I site. Second strand synthesis by DNA polymerase I followed RNase H treatment of the template. Blunt-ended cDNAs were ligated to *EcoR*I adapters, phosphorylated and digested with *Xho*I. cDNAs were then size fractionated over Sepharose and ligated into the Uni-ZAP XR vector arms. Following phage packaging, XL1-Blue MRF′ cells were infected to determine the library titre of 2.3 × 10^5^ pfu/ml on the basis of blue/white selection. Plaques from this primary titration were picked at random, insert sizes determined by PCR with M13F and M13R primers, and sequenced on an ABI9600 capillary DNA sequencer as part of a larger-scale EST analysis (Harcus, Y., Filbey, K., Blaxter, M. and Maizels, R., unpublished). The library was amplified before storage at a titre of 1 × 10^10^ pfu/ml. One of the first randomly-picked sequences corresponded to a full-length 156-aa open reading frame with significant similarity to *C. elegans* and mammalian C-type lectins. The ORF encoding the full-length mature protein (without predicted signal sequence) was amplified for expression of recombinant protein in pET21b in *E. coli* using the following primers : forward primer (*Nde*I site underlined) CGC CAT ATG AAT GAA TGT TGG TAT GTG (nt 67-84 of CDS); and reverse primer (*Xho*I site underlined) CCG CTC GAG AAT CTT ACA AAC GTA GCC (nt 468-451 of CDS). PCR products were ligated into pGEM-T, sequenced to verify no error had occurred, and plasmid DNA prepared for restriction digestion and insertion into pET21b. The cDNA sequence has been deposited in GenBank as FJ456978.

### 2.3 *N. brasiliensis* life cycle

The gastrointestinal nematode parasite *N. brasiliensis* was maintained in rats as described elsewhere [26,35]. Infective L3 larvae were collected from plates of charcoal-faecal culture; 4000 L3 were injected into SD rats, and lung-stage L4 collected 48 h later by Baermannisation. Adult parasites were taken 6 days post-infection.

### 2.4 *Nb*-CTL-1 isolation

A *N. brasiliensis* Uni-Zap phage library from M. Selkirk (Imperial College London) prepared from day 5 mixed-sex adults as described elsewhere [36], and multiple clones sequenced for EST analysis [36]. Cluster NBC00175 (http://www.nematodes.org/nembase3/cluster.php?cluster=NBC00175) was found to contain a 480-nt sequence with similarity to C-type lectins, and was designated *Nb*-CTL-1. Additional ESTs subsequently extended this cluster to 592 nt, but no start site was included. An identical cluster was also assembled as NB01234 at nematode.net. Primers made to isolate 5′ and 3′ ends by PCR with appropriate vector primers from the cDNA library were as follows : for 5′ end, YMH-L-R1 (223-202): GCC CTC CTT TAT CCT GGC AGC G ; for 3′ end, YMH-L-F1 (524-538): ATA TCC GTT GTG ACG CG (numbers relate to nt positions in final, full-length sequence). Because *Nb*-CTL-1 contains an internal *Nde*I site (nt 491-496 of ORF), an alternative expression system was employed. cDNA encoding recombinant *Nb*-CTL-1 protein (aa 21-191) was amplified with primers containing *BamH*I sites (underlined): YH-EXP-F (58-78): GGA TCC G ACA TTC AAG GCG ATA GCG CCG; YH-EXP-R (556-576, including stop codon): GGA TCC CTA AAC TGC GAC TTC GCA CAC. The amplicon was ligated into pGEM-T, colonies from transformed *E. coli* were picked and sequenced, and the appropriate plasmid preparation was digested with BamHI enzyme. Gel-purified inserts were ligated into pET15b, transformants were colony screened for orientation, and expression was induced as described below. The coding region cDNA sequence has been deposited in GenBank as FJ456979.

### 2.5 *Nb*-ctl-2 isolation

Searching nematode genome databases with the *Nb*-CTL-1 nt sequence, a related gene (cluster NB00604 at http://www.nematode.net) was identified which encoded a full-length homologue of the CTL family. Primers were made to amplify the coding sequence from the cDNA library, and the sequence of the amplified product were verified to match that of the original cluster. The confirmed coding region cDNA sequence has been deposited in GenBank as FJ456980.

### 2.6 Bioinformatics

Sequence analysis and manipulations were carried out with MacVector 9, and sequence database searches on the NCBI website. For searches against individual nematode species datasets, the Nembase3 (http://xyala.cap.ed.ac.uk/services/blastserver/) and Nematode.net (http://www.nematode.net/) websites were used. *C. elegans* data were retrieved from Wormbase ( http://www.wormbase.org/). Signal peptide cleavage sites were predicted with SignalP 3.0 [37].

### 2.7 RT-PCR

First strand cDNA was prepared from L3, lung L3, gut L4, male and female worms and eggs. In each case, approximately 50 µl of packed parasites were homogenised in 1 ml TRIzol (Invitrogen), and the aqueous layer recovered after centrifugation was mixed with isopropanol to precipitate RNA. The RNA pellet was washed in ethanol, air-dried, and treated with DNase (Ambion DNA-free kit). Reverse transcription was performed with MMLV reverse transcriptase (Stratagene) according to the supplier's instructions.

First strand cDNA was then used for conventional PCR using the following primers, and the programme of 5 °C for 5 min, 94 °C for 1 min, 60 °C for 1 min, 72 °C for 2 min for 28 cycles and 72 °C for 10 min. For *H. polygyrus* CTL-1-specific RT-PCR, the same primers were used as described in Section 2.1, complementary to nt 67-84 and 468-451 of the coding sequence. Species-specific actin primers were used as follows:

Actin 216-237: TGA GCA CGG TAT CGT CAC CAA C
Actin 918-897: TTG AAG GTC TCG AAC ATG ATC TG.

For *N. brasiliensis* additional primers were designed in order to distinguish the two newly recognised gene sequences, as given below; specific actin primers used are also shown.

*Nb*-CTL-1 80-105 GTA ATG ACG GAG GAT CGG CTC TTT AC and
*Nb*-CTL-1 584-558 TGT GCG AAG TCG CAG TTT AGC TTT GGA;
*Nb*-CTL-2 84-109 GGA TAT TAA CCC AGC TCT CAG GCA AC and
*Nb*-CTL-2 450-476 TAC GTT GTC ATA CGG TAG CAT GGA TCC
Actin BM279147 87-111 ACG ACG TGG CAG CTC TCG TTG TGG
Actin BM279147 406-382 GGT GCT TCG GTC AGC AGC ACG GGA.

### 2.8 Protein expression

*E. coli* BL21(DE3) bacteria were transformed with recombinant pET15b (*Nb*-CTL-1) or pET21 (*Hp*-CTL-1); cultures were then induced to express protein in the presence of 1 mM IPTG for 3 h at 37 °C. Pilot experiments had established that the proteins were insoluble in the absence of 8 M urea. Bacteria were therefore recovered by centrifugation and pelleted cells were taken up in binding buffer containing 8 M urea. This suspension was sonicated and the soluble supernatant was recovered following centrifugation. The supernatant was filtered through a 0.45 µm filter and applied to a nickel-chelating column (Amersham) on an Akta chromatography system. Recombinant proteins were recovered by elution with 0.5 M imidazole. As an unrelated control protein, *B. malayi* galectin [38] was expressed in a similar plasmid (pET29c) in the same *E. coli* host cells, and purified in the same way, except that the protein was soluble in the absence of urea. All three proteins contained a hexa-histidine tag to permit affinity chromatography by nickel-chelating columns.

### 2.9 Excretory–secretory (ES) antigens

*H. polygyrus* and *N. brasiliensis* excretory/secretory antigens (respectively termed HES and NES) were collected from adult worms as previously described [32] Briefly, *N. brasiliensis* worms were collected from rats 6 days post-infection and cultured for 7 days in serum-free RPMI-1640 medium containing 100 U/ml penicillin, 100 µg/ml streptomycin and 1% glucose. Supernatants collected between days 1 and 7 of culture were pooled and diafiltrated to a concentration of 1 mg/ml over a 10,000 mol.wt. cut-off Amicon filter. For *H. polygyrus*, similar procedures were used except that cultures of viable worms, collected from CF1 mice 14 days post-infection, were maintained for up to 21 days *in vitro* (Grainger, J. et al., submitted for publication).

### 2.10 Antibody production

Antibodies were made in male Sprague–Dawley rats, by primary immunisation with 300 µg recombinant CTL in alum adjuvant, followed by two further boosts of 50 µg CTL in alum at days 28 and 35. To prepare the immunogen, appropriate concentrations of CTL solubilised in 8 M urea were mixed with aluminium sulphate, and the precipitated protein was washed 3 times in PBS to remove traces of urea. High titre antisera were collected on d42, seven days following the final antigen boost.

### 2.11 ELISA

ELISA plates were coated with 1 µg/ml of HES, NES, r*Hp*-CTL-1 or r*Nb*-CTL-1 in 0.06 M carbonate buffer pH 9.6, overnight at 4 °C; plates were then blocked with 5% bovine serum albumin in the same buffer for 2 h at 37 °C, and washed 3 times in TBS-0.1% Tween 20 (TBS-T). Serum from normal or immunized rats were then diluted as indicated in the Figures and added to the appropriate wells for 75 min at 37 °C, before plates were washed 5 times and incubated with 1/2000 dilution of horseradish peroxidase (HRP)-conjugated rabbit anti-rat Ig (DAKO P450) in TBS-T for 1 h 37 °C. Following 4 washes in TBS-T and a final wash in detergent-free TBS, ABTS substrate (Kirkegaard and Perry) was added and absorbance read at 405 nm 15 min later.

### 2.12 Western blots

SDS-PAGE was carried out essentially as described previously [38] using Invitrogen NuPAGE 4–12% precast gradient gels; 10 µg of sample (or in the case of recombinant protein, 1 µg) was loaded per lane. After SDS-PAGE, gel-separated proteins were transferred electrophoretically to nitrocellulose at 45 mA per gel for one hour in Hoeffer Western blot buffer (10% methanol in NuPAGE transfer buffer, Invitrogen NP0006), then filters were blocked in 3% soya milk/TBS-T for 1 h and incubated with rat anti-*Nb*-CTL-1 (1/1000) or naive rat serum, diluted in the same blocking buffer, overnight at 4 °C. Filters were then washed in TBS-T three times (10 min each with rocking) and incubated with HRP-conjugated rabbit anti-rat Ig (DAKO P450) for a further 1 h in blocking buffer. Filters were washed again (3 times with TBS-T, 5 min each) and gels were developed using the Chemiglow kit (Alpha Innotech), with a 25-s exposure, as per manufacturer's instructions.

### 2.13 Immunofluorescence

Adult *H. polygyrus* worms were harvested from murine duodenum 14–28 days post-infection, washed and snap-frozen on dry ice into Cryo-M-Bed mountant (Bright Instruments). Sections of 5 μm thickness were cut onto Polysine™ slides using a Leica cryostat. Sections were air dried for 1 h prior to fixing in 100% acetone for 10 min. Sections were allowed to dry for > 15 min before either staining or storage at − 80 °C. For staining, sections were washed twice in PBS for 10 min at room temperature. Primary antibodies were diluted 1/100 in PBS containing 1% fetal calf serum. Sections were stained with 100 μl of the primary antibody (diluted 1/100 in PBS with 1% FCS) for 2 h at room temperature in a humidified box. Sections were then again washed twice in PBS for 10 min before application of TRITC-conjugated anti-rat IgG TRITC (Sigma) diluted 1:100 in PBS. Following a further 1 h incubation, slides were washed with two changes of PBS for 1 h, dried, and mounted with Vectashield^®^ (Vector Labs) before viewing on a fluorescence microscope.

## 3 Results and discussion

### 3.1 Sequences of *H. polygyrus* and *N. brasiliensis* CTL genes

**Fig. 1:**
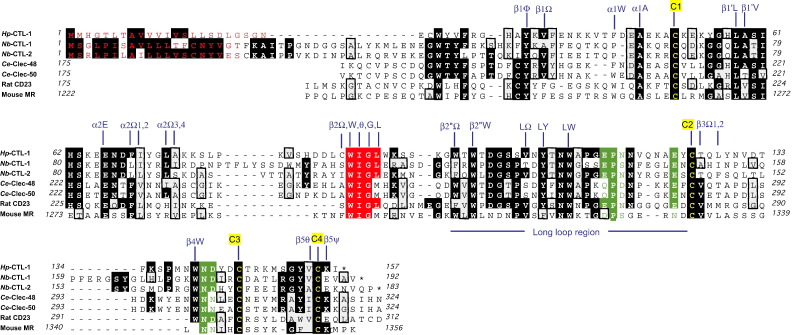
Sequence conservation of CTLs from trichostrongyloid nematodes. Sequences of C-type lectins (CTLs) from *H. polygyrus* and *N. brasiliensis*, compared with known sequences from two *C. elegans* homologues (CLEC-48 and CLEC-50), and two mammalian homologues (CD23, and MR or CD206). Accession numbers for *Hp*-CTL-1 and *Nb*-CTL-1 and -2 are FJ456978, FJ456979 and FJ456980FJ456979FJ456980 respectively. The *N. brasiliensis* CTLs correspond to clusters for *Nb*-CTL-1 designated NBC00175 at Nembase3 and NB01234 at nematode.net; and for *Nb*-CTL-2, NBC00697 at Nembase 3 and NB00604 at nematode.net databases. The C-terminal domains (aa 175-324) of both CLEC-48 and CLEC-50 are shown, as is also the case for rat CD23 (175-312, AAH78749). The 8th CRD of mouse MR/CD206 (aa 1222-1356, ABC87985) is shown. Predicted signal peptides of *Hp*-CTL-1 and *Nb*-CTL-1 and -2 are shown in red letters; for *Nb*-CTL-2, cleavage sites at 20/1 and 23/24 are of similar likelihood, but only the shorter signal peptide is indicated. The characteristic CTL motif WIGL is highlighted in red background. Residues implicated in calcium and sugar binding are shown in green letters or green background. Conserved cysteine residues are in yellow letters and numbered C1-C4. Residues involved in forming the hydrophobic core are labelled according to Zelensky [40], with prefixes indicating structural units (β sheets β1, β2, β3; alpha helices α1, α2; and Long loop region); suffixes indicate individual amino acid, or type of amino acid (ϕ, aromatic; Ω, aliphatic or aromatic; θ, aliphatic; ψ, charged). (For interpretation of the references to colour in this figure legend, the reader is referred to the web version of this article.)

*H. polygyrus* and *N. brasiliensis* are the two most widely-studied nematode parasites of rodents, and are natural parasites of the intestinal tract of mice and rats respectively. As part of a larger transcriptomic project on these species, we noted clones with significant sequence similarity to the C-type lectin family. From an adult *H. polygyrus* cDNA library, we identified *Hp*-CTL-1 as a full-length transcript encoding a 22-aa predicted signal peptide and a 135-aa single-domain CRD (Fig. 1), with a predicted mature mol.wt. of 17,811 before signal peptide cleavage is taken into account.

In the case of *N. brasiliensis*, two clusters of ESTs were identified from adult cDNAs, one of which required additional amplification with library vector primers to ascertain the complete sequence. In both cases, full-length sequences were verified from multiple PCR-derived clones from the cDNA library. The two genes, assigned *Nb*-CTL-1 and -2, encode predicted proteins of 191 and 183 aa respectively (Fig. 1). Both contained predicted signal sequences, in the first case of 20 aa, while in the second there was a similar likelihood of signal peptidase cleavage after 20 or 23 aa. The three CTL amino acid sequences were between 38–52% identical to one another. It was also noted that minor sequence variants were present in different clones from the cDNA library of *N. brasiliensis*, indicating some degree of polymorphism at these gene loci; similar minor variation has been noted with CTL sequences from the tissue-dwelling nematode *Toxocara canis* [39]. None of the proteins from *N. brasiliensis* or *H polygyrus* include potential *N*-glycosylation sites, which contrasts with the significant *N*-glycosylation (as well as O-glycosylation) of secreted CTLs of *T. canis* [18,19].

### 3.2 Similarities with C-type lectins

The full-length sequences were first used to search other nematode datasets for further homologues. The closest similarity was found to *C. elegans* CLEC-48 (for *Hp*-CTL-1 and *Nb*-CTL-2) and 50 (*Nb*-CTL-1) (Fig. 1), two highly homologous proteins which appear to have recently duplicated in the *C. elegans* genome. Interestingly, CLEC-50 is known to be expressed in the worm intestine and to be up-regulated during infection of *C*. elegans with the bacterial pathogen *Serratia marcescens* [22], indicating that this gene product acts in the innate immune defence system of the worm.

Further homologues were then identified in vertebrate databases. Indeed, the two most similar protein sequences in the entire NCBI non-redundant database to *Nb*-CTL-1 were CD206 the macrophage mannose receptor, and CD23, the low affinity Fcε receptor [10] (Fig. 1), and in the latter case the similarity was highest to the homologue from the rat, the natural host species for *N. brasiliensis*. For *Hp*-CTL-1, similarity to CD206 was also found although to a lesser degree than to lectin-like sequences from the snake *Liophis poecilogyrus* and the carp *Cyorinus carpio.* There were also similarities to other invertebrate lectins such as that from the sea anemone, *Nematostella vectensis*.

The three dimensional structures of many of the mammalian CTL family members (including CD206) have been resolved, and the role of many key residues determined [40]. The protein has two loops, stabilised by 2 disulphide bonds between C1–C4 and C2–C3, the latter holding in place a long protruding flexible loop which interacts with calcium and sugar ligands. Each of these cysteine residues is perfectly conserved in the new nematode lectins. Some 26 additional residues are implicated in forming the stable hydrophobic core of the protein (Fig. 1); some 21 of these are fully conserved in all 3 new sequences described here, including the characteristic WIG (or WIGL) motif (highlighted in red in Fig. 1). Thus, it seems likely that the strongylid proteins share a similar 3-dimensional structure to those known for mammalian CTLs. Importantly, the flexible loop of the newly-identified CTL homologues is of similar length to those of functional mammalian lectins, and not abbreviated as is observed in related proteins such as CD94 which have lost carbohydrate-binding capacity [5].

The key contact residues between CTLs, two Ca^2+^ ions, and the sugar ligand, have been determined for a number of mammalian lectins. For example an EPN motif (Fig. 1) is associated with mannose binding, while QPD is linked to galactose specificity; in addition, a glutamic acid residue adjacent to C2, and an Asn–Asp pair close to C3 are also involved. These positions are well conserved in the strongylid lectins, indicating that they may be functional sugar-binding proteins, although confirmation of this awaits experimental investigation with correctly-folded recombinant proteins.

### 3.3 Phylogenetic analysis

*H. polygyrus* and *N. brasiliensis* are within the same taxonomic superfamily (Trichostrongyloidea) as the sheep nematode *H. contortus,* which is currently the subject of complete genome sequencing. Both EST and genomic *H. contortus* datasets were searched using interfaces at Washington University and the Wellcome Trust Sanger Institute respectively. The same highly similar homologue was found in both transcriptomic and genomic sets (HC01174), which predicts a 171-aa full-length protein. In addition, more distantly related C-type lectins from a range of other nematode parasite species were noted. Interestingly, the new CTLs aligned with subsets of those in the free-living species *C. elegans*, *C. briggsae* as well as the marine nematode *Laxus oneistus* [23] .

**Fig. 2:**
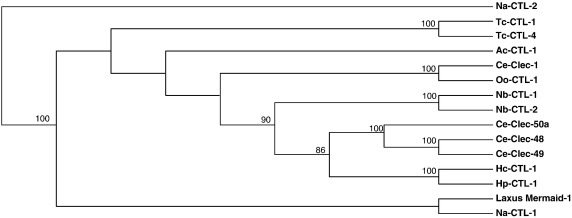
Phylogenetic relationship of nematode CTLs. The CRD domains of the following nematode CTLs were optimally aligned and analysed for phylogenetic relationships using MacVector UPGMA, best fit (with bootstrap values shown where > 50); systematic tie-breaking and Poisson correction. Species names and accession numbers or genome centre cluster identifiers: *Ancylostoma ceylanicum Ac*-CTL-1 (AF172652); *C. elegans Ce*-CLEC-48 (CBG05976, C14A6.1); *Ce*-CLEC-49 (WO4E12.6); *Ce*-CLEC-50 (WO4E12.8); *Haemonchus contortus Hc*-CTL-1 (Cluster HC01174 at nematode.net); *Hp*-CTL-1 (FJ456978); *Laxus oneistus* Mermaid lectin (AAX22004); *Necator americanus Na*-CTL-1 (AAY58318); *Na*-CTL-2 (AF388311); *Nb*-CTL-1 (FJ456979); *Nb*-CTL-2 (FJ456980); *Ostertagia ostertagi Oo*-CTL-1 (cluster OS00829 at nematode.net); *Toxocara canis Tc*-CTL-1 (AF041023); and *Tc*-CTL-4 (AF126830).

Phylogenetic analysis of existing and newly-identified nematode parasite CTLs, together with the *C. elegans* CLEC proteins, showed that within-species similarities are common (indicating recent diversification within each lineage), and also a close relationship between *H. polygyrus* and *H. contortus* CTLs, supported by bootstrap analysis (Fig. 2). With the growing number of known nematode CTL sequence, and with better ability to discriminate between true orthologues and paralogues, the phylogeny of this gene family should become increasingly well-resolved.

### 3.4 Expression is predominantly by the adult stages

**Fig. 3:**
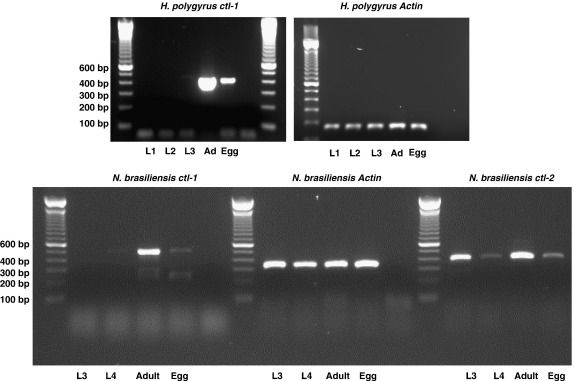
CTL gene expression primarily by adult stages. RT-PCR was performed on first strand cDNA from L1, L2, L3, adults and eggs of *H. polygyrus* (top panels), using primers specific for *Hp*-CTL-1 (left) or β-actin (right). Similarly, cDNA from *N. brasiliensis* L3, lung L4, adults and eggs was used as a template for *Nb*-CTL-1 lower panel, left), *Nb*-CTL-2 (right) or β-actin (centre) primers appropriate for each species of parasite. For *Nb*-CTL-1/2 amplifications, products were excised and sequenced to verify amplification of the correct transcript.

For both *H. polygyrus* and *N. brasiliensis*, the original CTL-encoding ESTs had been isolated from adult worm cDNAs, and expression of a *ctl* gene by the related human hookworm *Necator americanus* has previously been shown to be restricted to the adult stage [41]. To investigate expression at different points in the life cycle of these parasites, RT-PCR was employed with cDNA prepared from larval (free-living) and adult (intestinal) stages and eggs, as well as (for *N. brasiliensis)* the intermediate larval forms from the lung of rats. To distinguish *Nb*-CTL-1 and *Nb*-CTL-2 mRNA, gene-specific primers were designed, and in each case products of the PCR reactions were sequenced to verify authenticity of the amplicons. The results (Fig. 3) show that lectin gene expression is primarily restricted to the adult, intestinal-dwelling, stages of both species, although there are differing degrees of expression by immature stages. Thus, both *Hp*-CTL-1 and *Nb*-CTL-1 are present only in the adults and eggs, with the latter showing considerably lower levels of transcript. However, *Nb*-CTL-2 mRNA is present in all stages tested, including the free-living L3 form.

Because the L3 of *N. brasiliensis* is a transmission stage in readiness to penetrate mammalian skin, expression at this point does not preclude a functional role in interacting with the host immune system. In all cases, expression by the gut-dwelling adults also raises the possibility that these gene products fulfill a function in this key interface with the mammalian host. However, an alternative possibility is that gut-dwelling nematodes utilise CTL proteins to counter resident bacterial populations, in a manner analogous to the antibacterial defence functions of homologues in *C. elegans* [42].

### 3.5 Antibodies to CTLs react with ES proteins and with CTLs from both species

**Fig. 4:**
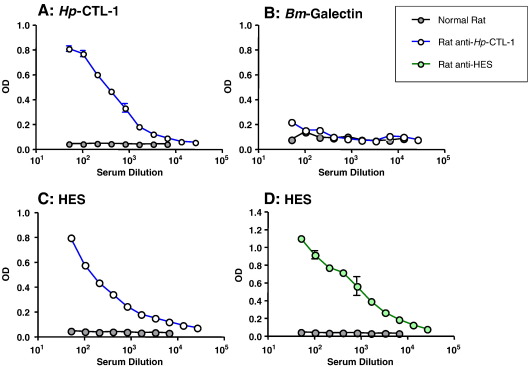
Antibodies to *Hp*-CTL-1 react with *H. polygyrus* excretory**–**secretory antigens (HES). Polyclonal antibodies raised in rat against recombinant *Hp*-CTL-1 were tested by ELISA for reactivity with the homologous immunogen *Hp*-CTL-1 (A), an unrelated recombinant protein containing a hexa-histidine tag, *Bm*-Galectin (B), and HES (C). Normal rat serum controls are shown in each panel. (D) Positive control of rat anti-HES serum tested by ELISA on HES. Data shown are means ± SD of duplicate determinations at each dilution, and similar results were obtained with two different polyclonal antisera raised against *Hp*-CTL-1.

Recombinant *Hp*-CTL-1 and *Nb*-CTL-1 proteins were produced in *E. coli,* but as reported for other nematode CTLs ([19]) proved to be insoluble in aqueous buffer, indicating mis-folding. Proteins were solubilised in urea and alum-precipitated for immunisation of rats, or added directly to ELISA coating buffer. Rat anti-*Hp*-CTL-1 antibodies bound the recombinant protein (Fig. 4A), but not an unrelated protein (*Bm*-Galectin) produced in the same bacterial expression system (Fig. 4B). Significantly, anti-*Hp*-CTL-1 antibodies reacted strongly to the secreted protein fraction of *H. polygyrus*, HES (Fig. 4C), although to a lesser degree than anti-HES antiserum (Fig. 4D). However, anti-HES antibodies did not bind to the recombinant protein, which may reflect the fact that the incorrectly folded *Hp*-CTL-1 antigen used in ELISA lacks the conformational epitopes of the native protein. Both native and recombinant proteins, however, would share linear epitopes, allowing antibodies to misfold recombinants to recognise the native antigen.

**Fig. 5:**
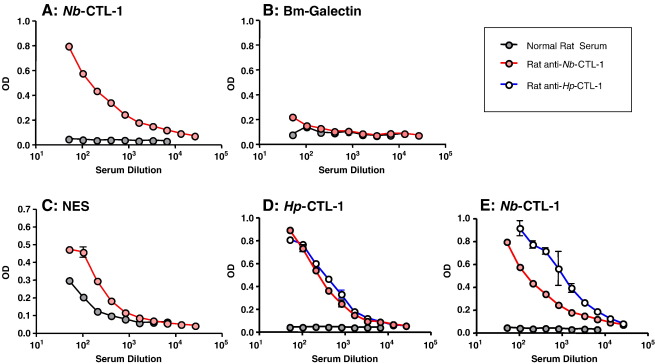
Antibodies to *Nb*-CTL-1 react with *N. brasiliensis* excretory**–**secretory antigens (NES), and cross-react between the two species. Polyclonal antibodies raised in rats against recombinant *Nb*-CTL-1 were tested by ELISA for reactivity with the homologous immunogen *Nb*-CTL-1 (A), an unrelated recombinant protein, *Bm*-Galectin (B), and NES (C). Normal rat serum controls are shown in each panel. Sera raised against *Hp*-CTL-1 and *Nb*-CTL-1 were compared for reactivity with *Hp*-CTL-1 (D) and *Nb*-CTL-1 (E), showing substantial cross-reactivity. Data shown are means ± SD of duplicate determinations at each dilution, and similar results were obtained with two different polyclonal antisera raised against each antigen.

Anti-*Nb*-CTL-1 antibodies were also found to react strongly to the homologous recombinant (Fig. 5A) and not to the control protein (Fig. 5B). Reactivity to *N. brasiliensis* secretions (NES) was also found (Fig. 5C) although less convincingly than with the equivalent experiment with HES (Fig. 4C). It was also found that antisera to either *Hp*-CTL-1 or *Nb*-CTL-1 cross-reacted with the other lectin (Fig. 5D, E). The two lectins shared 38% aa identity (55/143 positions); hence it is likely that the same antibodies would also recognise *Nb*-CTL-2 (52% aa identity, 85/163).

### 3.6 Secretion of C-type lectins by adult parasites

**Fig. 6:**
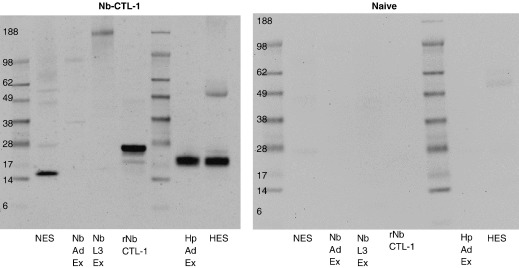
Western blot analysis identifies a 19-kDa protein in HES and a 15-kDa protein in NES reactive with anti-CTL antibodies. Polyclonal rat antiserum, raised to *Nb*-CTL-1 and cross-reactive with *Hp*-CTL-1 (see Fig. 5) was used to probe Western blots of SDS-PAGE separated nematode antigens (left hand panel). Normal serum from a naive rat was used as a negative control (right hand panel). In each panel, the following antigens were tested : adult *N. brasiliensis* excretory–secretory antigen (NES); somatic extracts of *N. brasiliensis* adult (Nb Ad Ex) and infective larval (Nb L3 Ex) stages; recombinant *Nb*-CTL-1 protein (rNb CTL-1); somatic extract of *H. polygyrus* adult worms (Hp Ad Ex), and adult *H. polygyrus* excretory–secretory antigen (NES). Unmarked lanes contain marker proteins of 6–188 kDa. Proteins were separated on a one-dimensional 4–12% SDS-PAGE gel.

To identify protein products of the CTL genes, the reactive antisera tested above were used in Western blots of various preparations of *H. polygyrus* and *N. brasiliensis*. Extracts and secretions (HES, NES) were separated by 1-D SDS-PAGE, and blotted proteins probed with anti-CTL serum or normal rat serum (Fig. 6). Strong reactivity was observed to a single 19-kDa band in somatic extracts or secretions (HES) of *H. polygyrus*; this size is larger than the predicted mol.wt. of *Hp*-CTL-1 following cleavage of the signal peptide (15.6 kDa), but is within the range accountable by post-translational modifications (such as O-glycosylation) and/or anomalous gel migration. These factors are thought to account for the 32-kDa apparent mol.wt. of *T. canis* CTL-1, which is encoded by a polypeptide with predicted mol.wt. of 21.5 kDa [18]. With *N. brasiliensis*, a 15-kDa NES component was identified, which in this case is smaller than the 19.5 kDa prediction for the unmodified protein.

An inherent problem with large multi-gene families such as the CTLs, is that closely-related gene products may not be distinguished by antibody binding. Hence, while our data do not unequivocally identify CTL-1/2 as constitutents of HES and NES, it can be concluded that either these or very closely-related proteins are secreted by adult stage of both parasites. Moreover, the RT-PCR data showing selective up-regulation of the CTL-1/2 genes in adult worms, shows that each gene follows an expression pattern consistent with secretion by the adult parasites.

### 3.7 Localization of C-type lectin to the cuticle of adult *H. polygyrus*

**Fig. 7:**
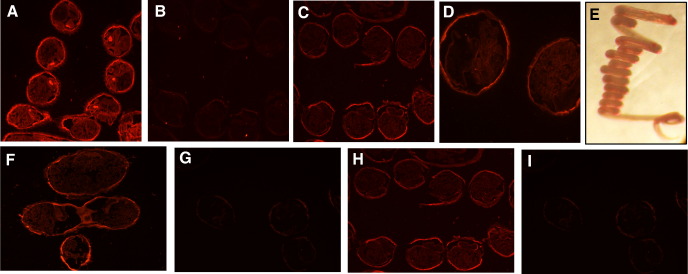
Immunofluorescent binding of anti-*Hp*-CTL-1 antibodies to the cuticle of adult male and female *H. polygyrus*. A. Positive control antiserum (rat anti-HES), stained with TRITC-conjugated anti-rat Ig stain, ×200; B. Negative control normal rat serum (double exposure time compared to A), ×200 C. Anti-CTL-1 (same exposure as B), ×200 D. Anti-CTL-1, ×400 E. Light micrograph of coiled adult *H. polygyrus*, showing how closely-spaced sections represent succesive portions of same worm, ×40 F,G. Female, anti-CTL-1 (F) and normal rat (G), ×200 G,H. Male, anti-CTL-1 (H) and normal rat (I), ×200.

To localize CTL expression within the parasite, adult *H. polygyrus* worms subjected to immunofluorescent staining with polyclonal anti-*Hp*-CTL-1 rat antiserum. The results, shown in Fig. 7, revealed preferential binding to the adult cuticle. A similar finding was reported for *T. canis* CTL-1 (TES-32), which is both secreted by larval parasites of this species, and localized to the cuticle by immuno-electron microscopy [43]. However, it is not known whether, in the case of *H. polygyrus* adult worms, secretion takes place as is thought to occur in *T. canis* larvae. To establish whether *Hp*-CTL-1 was differentially expressed by male and female adult worms, individuals of either gender were tested by immunofluorescence, and showed equal intensity of cuticular staining with anti-CTL-1 antiserum (Fig. 7E, G).

## 4 Conclusion

The C-type lectin domain is an ancient and versatile evolutionary unit which is widely expressed throughout the metazoa. In *C. elegans* alone, there are over 270 potential CTL gene products, while the recently-completed genome of the plant-parasitic nematode *Meloidogyne incognita* encodes over 50 homologous genes [44]. Even within ~ 130 ESTs taken from a single tissue (the intestine of *H. contortus*), 9 CTL-like transcripts were found [45], indicating that many more family members may exist. These proteins may act in many different biological systems, from developmental to structural, and many members of the broader CTL superfamily no longer show sugar binding but have evolved alternative functions on the basis of the same general molecular scaffold [2,40,46].

Interestingly, it is already established that within the single Nematode phylum CTL proteins in different environments appear to mediate very different interactions with other organisms. In the free-living *C.elegans*, CTLs such as CLEC-50 are defence proteins against bacterial infection [22,47], while in the marine Stilbonematodes the Mermaid lectin captures symbiotic bacteria [23]. The release of CTLs by infective stages of parasitic species has led us to postulate they act in an “offense” capacity, perhaps by interfering with normal lectin–glycan interactions of the host immune system [20]. This proposition remains conjectural, although some parasite secreted lectins are known to bind host ligands, as in the CTL-4 lectin elaborated by tissue-dwelling *T. canis* larvae [19]. An intriguing possibility is that the lectins of gastrointestinal nematodes may interact with the complex mucin oligosaccharides which are known to alter significantly in response to worm infection [48]. It should also be noted that other parasite lectins appear to play physiological roles within the parasite, for example that from *Ancylostoma ceylanicum* [49], which is a functional Glc-NAc-binding lectin and most highly expressed in male gonads and sperm, indicating that its function is in worm reproduction.

Our report now extends the spectrum of known parasite CTL-like proteins, and by demonstrating secretion, suggests that lectin-like activity may be a more general feature of the nematode–host interface. The origin of the secreted lectins remains to be determined, and it will be important to distinguish between products released, for example, in the nematode digestive tract, and those which may be directed for secretion into the host environment. Functional testing of these new gene products, by expression of authentically folded proteins in eukaryotic expression vectors, will ascertain if indeed they are able to interfere with host recognition and/or effector processes.
